# Assessing the disease burden of Yi people by years of life lost in Shilin county of Yunnan province, China

**DOI:** 10.1186/1471-2458-9-188

**Published:** 2009-06-17

**Authors:** Shang-Cheng Zhou, Le Cai, Chong-Hua Wan, Yi-Ling Lv, Peng-Qian Fang

**Affiliations:** 1 School of Medicine and Health Management, Tongji Medical College, Huazhong University of Science and Technology, Wuhan 430030, PR China; 2 Faculty of public administration, YunYang Medical College, Shiyan 442000, PR China; 3 Faculty of Public Health, Kunming Medical College, Kunming 650031, PR China

## Abstract

**Background:**

Years of Life Lost (YLL) is one of the methods used to estimate the duration of time lost due to premature death. While previous studies of disease burden have been reported using YLL, there have been no studies investigating YLL of Yi people in rural China. Yunnan Province ranks first in terms of Yi people in China. This paper uses YLL to estimate the disease burden of Yi people in Shilin county of Yunnan Province. This study aims to address the differentials about YLL between Yi people and Han people for providing useful information for health planning.

**Methods:**

We applied the Global Burden of Disease (GBD) method created by WHO. YLL rate per 1,000 were calculated from medical death certificates in 2003 in Shilin Yi Nationality Autonomous County (Shilin county).

**Results:**

The male had greater YLL rate per 1,000 than did the female almost in each age group. It demonstrated a higher premature mortality burden due to injuries in Shilin county. Among the top non-communicable diseases, respiratory diseases are the most common mortality burden. Yi people are still suffering from maternal conditions, with two times the burden rates of Han people. For Yi people, while malignant neoplasm was one of the least burden of disease for male, it was the greatest for female, which is the opposite to Han people.

**Conclusion:**

Strategies of economic development should be reviewed to enhance the prevention and treatment of injuries, maternal conditions and respiratory diseases for Yi people.

## Background

The Yi nationality has a total population of over 7,762,272 (male 3,989,391 and female 3,772,881) [1]. The members of Yi nationality are distributed in the west of the Yunnan-Guizhou Plateau and the southeast border region of the Qinghai-Tibet Plateau in Yunnan, Sichuan and Guizhou provinces and the Guangxi Zhuang Autonomous Region. Accounting for 61% of the total Yi population in China, Yunnan Province ranks first in terms of Yi population[2]. The mean mortality rate of Yi nationality is 7.89‰ (male 8.52‰, female 7.22‰)[1]. Shilin Yi Nationality Autonomous County (Shilin county) is a rural region in the southeast of Kunming City, Yunnan province[3,4]. It is 78 km from the main urban area of Kunming and covers a land area of 1,719 km^2^. Its permanent population amounts to 230,548 (116,204 males and 114,344 females) in 2003, wherein Yi population account for 34%[5]. In other study, chronic diseases have not displaced but added to the mortality burden from infectious and perinatal problems, and this double burden is a major challenge for health systems in Shilin county[6].

While a descriptive study on disease burden of certain nation in a given area is useful for planning, it would be more informative to see the full view of disease spectrums. Few studies about premature mortality burden have reported the differentials between Yi population and Han population. In some research articles, to understand the mortality model and expectant lifespan among the residents of main nationalities, indoor investigation was carried out for the people died in a period[7]. Therefore, we think that there may be some differentials from the disease burden of different nationalities between Yi people and Han people. Over the last 20 years, the measurement of population health status has received growing attention stimulated by the Global Burden of Disease (GBD) project[8]. Disability adjusted life year (DALY) enables researchers to combine Years of life lost (YLL) from premature death and years of life lived with disabilities (YLD) in a single indicator[9].

YLL is one of the methods to estimate the duration of time lost due to premature death, and is the mortality component of DALY. The conceptual and computational details of years of life lost have been presented elsewhere[10]. The YLL measure not only considers the number of deaths, but also takes into account the age at which death occurred. It is therefore a better tool for quantifying the burden of premature mortality compared to mortality rate.

In order to estimate the disease burden of Yi population and Han population, the present authors decided to carry out the burden of disease study. As the first step in identifying the disease burden of Yi population, the burden of Yi population caused by YLL was estimated. The results are presented in this paper.

## Methods

### Study populations

Shilin county was selected as the study region. Shilin county is a rural region with a population of 230,548 (116,204 males and 114,344 females) including 77,519 Yi people in 2003. All individuals residing and dying of Han population and Yi population in 2003 were included in the analysis.

### Data source

Causes of death were based on medical death certificate information, maintained by Shilin maternal and children hospital. All death reports were grouped by underlying cause of death as defined in the GBD study[11] and coded using the International Classification of Diseases, 9th revision (ICD-9) coding system.

In order to avoid some misreporting of age at death, and misclassification of cause of death, all medical death certificates were verified on the underlying cause of death by a team of two independent physicians. Any discrepancies were reviewed to obtain a consensus. All deaths assigned to ill-defined conditions were redistributed to other more defined causes according to the age and gender distribution of specific conditions, following the conceptual approach in the GBD study[12].

Overall mortality in Shilin county was divided into three broad groups of causes: Group I, communicable, maternal, perinatal and nutritional deficiencies; Group II, non-communicable; and Group III, all injuries. These were then further subdivided into several more specific causes[12]. Age was divided into some groups: 0~, 5~, 15~, 25~, 35~, 45~, 55~, 65~, 75~, 85 years and over or 0~, 5~, 15~, 30~, 45~, 60~,70~ and 80 years and over or 0~ 15~ and 60 years and over for the different needs. Overall age-specific mortality for each sex was plotted for visual comparison.

### Calculation of YLL

Premature mortality was estimated in terms of YLL[13].

The formula for YLL is:



where *K *is the age-weighting modulation factor, *C *is the age-weighting correction constant, *r *is the discounting rate, *a *is the age at death, *β *is a parameter from the age-weighting function, and L is standard life expectancy at age *a *from the national life table.

In the GBD study, YLL incorporated an age-weighting factor that takes into account the higher social value given to young adults in most societies, and added a discounting factor (social time preference) to reflect the fact that most individuals prefer benefits now rather than in the future[14]. YLL includes age weighting of the form: *Cxe*^-*βx*^, where *C *is a constant included so that the incorporation of unequal age weightings will not change the total estimated YLL burden, and *β *is a parameter that controls the shape of the age-weighting function, such that the maximum value of the function is reached at 1/*β *(for the GBD study, *β *was assigned a value of 0.04, so that the maximum value is at 25 years of age). When *K *is set equal to zero, the age weights are equivalent at all ages.

Consistent with the standard GBD approach, *C *was assigned a value of 0.1658 (this parameter controls the maximum height). This study calculated YLL with a 3% discounting rate per year. As China traditionally values living years in elderly and children, age weighting was not used in this study, so *K *was assigned a value of 0. To maintain comparability with other studies, YLL was calculated using the life tables provided in the GBD study, the model life table, West Level 26[15]. In this table, life expectancy at birth is 82.5 years for females and 80 years for males. To calculate YLL, the GBD DALY template was used[16]. This is a Microsoft Excel spread sheet that contains the formula to calculate YLL.

### Ethical approval

This study was approved by the Ethics Committee of Kunming Medical College, before carrying out the research.

## Results

**Figure 1:**
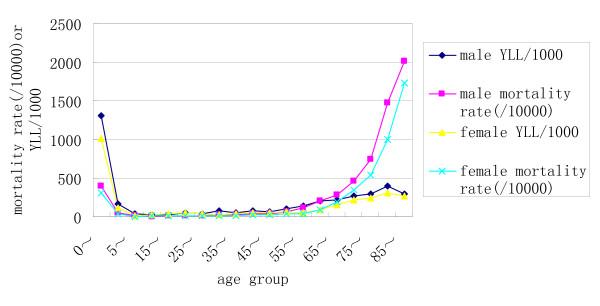
**Comparison of age-specific mortality rate and YLL/1000 by sex in Shilin county (2003)**.

The average life expectancy at birth was 70.8 years (95%CI: 70.0–71.5) in Shilin county according this survey data. There were 1,065 deaths (53.4% males, 46.6% females). The overall age-specific mortality rates and YLL/1000 by sex in Shilin county are illustrated in Figure 1. Mortality rates and YLL/1000 were higher by male throughout the whole age range. The old people had the highest mortality rate in age group of 80 years and over, whereas YLL/1000 was the highest in the 0~ years age group.

Table 1 showed distribution of death population by age and sex in Shilin county. Death population was the highest in the 70~ years age group. In general, Death men population had slightly higher values than women with the exception of the 80 years and over group.

**Table 1:** Distribution of death population by age, sex in Shilin county (2003)

	**Yi population**		**Han population**	
	
**Age group**	**Male**	**Female**	**Male**	**Female**
0~	13	9	23	12
5~	2	4	15	5
15~	5	5	25	23
30~	18	5	43	20
45~	28	16	52	35
60~	20	21	51	29
70~	42	33	111	99
80~	18	30	86	138
Total	146	123	406	361

Table 2 compared YLL/1,000 population by sex and age between Yi population and Han population in Shilin county. People aged 80 years and over were responsible for the highest years of life lost compared to the other age groups in each of the two nations. Children aged less than 5 years also had relatively high premature mortality burden in the two nations. In the age group of group 70~, Yi population had the lower value of YLL, whereas in the 0~ years age group, Han population was responsible for the lower premature mortality burden.

**Table 2:** YLL/1,000 population by age, sex in Shilin county (2003)

	**Yi population**		**Han population**	
	
**Age group**	**Male**	**Female**	**Male**	**Female**
0~	129.9	94.7	123.4	73.2
5~	17.1	22.7	45.2	16.5
15~	17.3	18.2	40.7	45.0
30~	55.7	17.6	54.4	31.1
45~	97.7	59.8	71.6	55.0
60~	89.7	102.2	101.1	66.5
70~	217.6	244.7	277.4	310.0
80~	214.7	186.3	207.1	188.8
All age *	57.8	42.5	67.0	51.3

*age adjusted

Calculating YLL by gender revealed male and female variation in the burden of disease (Table 3). For Yi population, the ranking of the top four disease groups in terms of YLL was the same for both genders with the exception of females for whom malignant neoplasms was the greatest burden of disease and Unintentional injuries were the sixth burden. For Yi people, while malignant neoplasms were one of the least burdens of disease for male, it was the greatest for female, which was opposite to Han people. For Han population, the ranking of the top six disease groups in terms of YLL was the same for both genders. The intentional injuries were also a much bigger burden for females than they were for males. The Unintentional injuries were also a much bigger burden for males than they were for females. Overall, there was a higher loss in males compared with females in YLL lost for either Yi population or Han population.

**Table 3:** YLL/1,000 population by sex and broad disease groups in Shilin county (2003)

**Disease groups**	**Yi population**		**disease groups**	**Han population**	
			
	**Male**	**Female**		**Male**	**Female**
Unintentional injuries	10.5	2.8	Unintentional injuries	16.4	8.3
Maternal conditions	9.1	5.5	Intentional injuries	9.2	11.5
Respiratory diseases	9.0	7.0	Respiratory diseases	9.1	10.2
Cardiovascular diseases	6.4	6.9	Malignant neoplasms	8.1	2.7
Intentional injuries	5.9	2.8	Cardiovascular diseases	5.7	8.1
Digestive diseases	4.6	1.5	Maternal conditions	5.5	4.2
Malignant neoplasms	3.8	7.7	Infectious and parasitic diseases	2.6	0.8
Respiratory infections	3.7	5.3	Congenital anomalies	2.6	1.4
Infectious and parasitic diseases	1.7	2.8	Neuro-psychiatric conditions	2.1	0.8
Neuro-psychiatric conditions	1.3	1.3	Genito-urinary diseases	1.4	0.7
Genito-urinary diseases	0	0.2	Digestive diseases	1.1	0.1
Congenital anomalies	0	1.0	Respiratory infections	1.0	0.6

Table 4 presented age breakdown of broad cause groups YLL/1,000 population of Yi population between male and female. It average accounted for the highest YLL/1,000 population in the 60 and over age group either male or female. The premature mortality from group I was the highest in the 0 age group. Non-communicable diseases were responsible for the highest YLL/1,000 population in the above age group. The premature mortality from injuries was the highest in young adults aged 15~ years. When the rate per thousand population was used, male had the higher premature mortality burden in all three death cause groups with the exception of male for whom were similar with female in 15 age group for group I and 60 and over age group for group II.

**Table 4:** YLL/1,000 population by age, cause and sex of Yi population in Shilin county (2003)

	**Male**				**Female**			
	
**Age group**	Group I*	Group II*	Group III*	**Total**	Group I*	Group II*	Group III*	**Total**
0~	21.02	21.58	22.42	65.02	12.18	15.15	10.84	38.17
15~	1.46	24.51	26.09	52.06	1.71	16.47	18.86	37.04
60~	2.99	155.66	9.14	167.79	1.62	157.27	6.3	165.19

* Group I: communicable, maternal, perinatal and nutritional deficiencies

Group II: non-communicable diseases

Group III: injuries

## Discussion

This study is the first analysis that has attempted to use YLL to measure the disease burden of Yi population in rural China. While local public health departments continuously monitor the health status of Yi population, this is the first time that YLL has been used to describe mortality patterns. This paper has presented data relating to the mortality burden of disease and injuries for Yi population in rural China and to compare it with Han population. YLL was calculated as the first step in estimating the burden of disease and injuries because the data for the calculation of YLL were readily available and can be considered as sufficiently reliable to enable the estimation of the burden caused by premature deaths. For the purpose of calculating YLD, data for Shilin county were not available. For this reason, the estimation of the total burden for selected disease was taken forward in the second phase of the study.

These findings indicated that the epidemiologic transition was well under way in the study region. As expected, the mortality burden was greater in men, either when deaths or YLL were considered. The old people had the highest mortality rate in age group of 80 years and over, whereas YLL/1000 was the highest in the 0~ years age group. Although calculating and explaining YLL is more complex than simple mortality rates, it adds value in demonstrating the effect on the population for each individual cause of death[17]. Mortality statistics tend to emphasize causes of death among the elderly, where most deaths occur, and thus give less priority to younger age groups[18]. YLL rank ordering tends to emphasis those causes of death, which often exist in younger age groups because of their larger future losses. Yi population had the lower value of years of life lost for the elders, whereas for infants Han population was responsible for the lower premature mortality burden. One of the explanations for these discrepancies could lie in different life style between the two nationalities compared.

This study suggested that the disease of maternal condition in terms of YLL/1000 population was higher for men than for women both in Yi people and Han people. The possible causes may be the underreporting deaths of men less than women because of the Chinese traditional concept "men are superior to women". Underreporting of deaths has been shown to be more common in infant deaths, especially women. Moreover, the number of the infant population in 0~ group was small, but larger in YLL of this age group for the infant population losing more life years. On the contrary, the individual in older group 80 and over loses less life years. However, older groups of 70~ and over 80y was still high in YLL for the large capability for these groups' high crude death rate. The information above was confirmed that deaths at younger ages may be considered of greater public health concern than deaths at older ages. If intervene steps to bring down the mortality rate of the infant population are performed, the YLL of population will descend sharply.

This study demonstrated a higher premature mortality burden due to injuries in Shilin county. Shilin county is mainly developed by tourism bringing a large number of visitors. Sharing of the road by high speed vehicles and walking villagers or visitors in addition to indirect acting factors of road traffic accidents may be an explanation for the heavy injuries burden [19-22]. The leading mortality burden in our study region includes unintentional injuries, respiratory diseases. Yi population was still suffering from maternal conditions, with two times the burden rates of the Han population, especially for female. Among the top non-communicable diseases, respiratory diseases were the most common mortality burden in both Yi and Han population. Cardiovascular diseases followed as the second highest burden in Yi population. Neuro-psychiatric conditions, although having a lower rate, still posed an important mortality burden. Moreover, this study suggests that the health priority areas of Yi people, relevant to the mortality burden, should include diseases during perinatal period and digestive diseases.

For Yi population, the leading causes of premature death were non-communicable diseases (group II). When the rate per thousand population was used, male had the higher premature mortality burden in all three death cause groups with the exception of male for whom were similar with female in 15 age group for group I and 60 and over age group for group II. This may be partly explained by the higher risk status, more unhealthy life style and bad access to health care for men. The above information indicated that with infectious diseases and prenatal problems resolved it would lead to a higher life expectancy and therefore more chronic diseases.

The causes of different disease burden between Han people and Yi people may be such as excessive drinking, hereditary factors, and so on. The phenomenon that ethnic minorities had more frequent reported alcohol intake than Han majority has been demonstrated in other studies[23]. These results thus linked culture only with alcohol and not with other disease risk factors.

Many investigators had emphasized that YLL rates provide a more complex measure of the impact of premature mortality than traditional death rates [24-26]. Compared with death rates, YLL gives greater proportional weight to those conditions that affect young people and less proportional weight to conditions affecting the elderly. Consequently, the ranking of some diseases by YLL differs from their ranking based on number of deaths of simple mortality rate.

There were a number of limitations to the present study. The strength of this study depends on the complete vital registration systems. Since Shilin county is surveillance point for vital statistics in China, the problem of underreporting of deaths found in many studies was thus minimized. Underreporting of deaths had been shown to be more common in infant deaths in a previous study, especially in rural regions[27]. Such work will be the focus for future researches. More detailed works could be done to estimate YLL at specific disease level that would take account of differences in mortality outcomes. Problems identified solely on the basis of mortality data may be underestimated. For example, in a study in Pakistan, injuries ranked eleventh according to YLL but second according to YLD, resulting in their ranking fifth based on DALY[28]. It is necessary to take next step to mention how to contribute DALY by YLL in the present study.

## Conclusion

The findings suggested that a strong health advocacy should be applied to Yi population in Shilin county, especially on maternal conditions and group III injuries. A continual and consistent effort in prevention and measures to reduce the burden from unintentional injuries in Shilin county should be strengthened.

## Competing interests

The authors declare that they have no competing interests.

## Authors' contributions

SCZ carried out the study and drafted the manuscript. LC and PQF conceptualized the research idea, participated in the design of the study. CHW and YLL interpreted the results and helped to draft the manuscript. All authors have read and approved the final version.

## Pre-publication history

The pre-publication history for this paper can be accessed here:


